# Misalignment and tilt effect on aspheric intraocular lens designs after a corneal refractive surgery

**DOI:** 10.1371/journal.pone.0243740

**Published:** 2020-12-14

**Authors:** Jesús Pérez-Gracia, Francisco J. Ávila, Jorge Ares, Juan A. Vallés, Laura Remón

**Affiliations:** Departamento de Física Aplicada, Universidad de Zaragoza, Zaragoza, Spain; Nicolaus Copernicus University, POLAND

## Abstract

**Purpose:**

To numerically evaluate and compare the tolerance to misalignment and tilt of aspheric intraocular lenses (IOLs) designed for three eyes: with standard cornea and with simulated corneas after myopic and hyperopic laser ablation surgery.

**Methods:**

Three aspheric IOLs of +20.00 diopter (D) with different spherical aberration (SA) ($Z_{4}^{0}$) values have been designed using a theoretical model eye. Drastic changes on the theoretical eye anterior corneal asphericity have been performed to simulate myopic and hyperopic refractive surgeries. The effect of IOL misalignment and tilt on the image quality has been evaluated using a commercial optical software design for the three eye models. Image quality was assessed from the modulation transfer function (MTF), root mean square (RMS) values of defocus, astigmatism, coma and spherical aberration ($Z_{4}^{0}$), and retinal images obtained from a visual simulator using an aleatory optotype of 0.00 LogMar visual acuity (VA).

**Results:**

IOL misalignment and tilt reduced MTF values in general, and increased wavefront aberrations errors. Aberration-free IOLs maintained best the MTF values when misalignments were applied, together with good on-axis optical quality. IOLs with negative SA ($Z_{4}^{0}$) correction decreased the MTF value under 0.43 for misalignments values higher than 0.50 mm with the three corneas. The effect of misalignment on RMS astigmatism and coma was correlated with the IOL SA ($Z_{4}^{0}$) and with the three corneas.

**Conclusions:**

This theoretical study shows that the largest degradation in image quality arises for the IOL with the highest amount of spherical aberration ($Z_{4}^{0}$). Moreover, it has been found that the aspherical design has a more influential role in misalignment tolerance than in tilt tolerance.

## Introduction

Since the first intraocular lens (IOL) was implanted in 1949 [1], optic and haptic designs of the IOLs have been evolving as new materials appeared. The development of soft materials changed the cataract surgery praxis, due to the possibility of introducing the lens inside the eye through a small incision [2, 3]. Nowadays, cataract surgery has become a surgical procedure that not only seeks to replace the cataractous lens but also to correct any refractive error and presbyopia; for this reason, new optical lens designs have been developed such as toric IOL, multifocal and accommodative IOLs, and aspheric optic designs. However, in this type of IOLs, the biomechanical stability inside the capsular bag is the key feature leading to a successful surgical procedure [4–6]. Misalignment, tilt or rotation affects the optical performance and efficiency of these IOLs, resulting in significant visual disturbances. Material properties [7], haptic designs [8], and the overall diameter of the IOL [9] are considered to be very important factors affecting the postoperative IOL stability.

There are different aspheric IOL designs in the market, which induce different spherical aberration ($Z_{4}^{0}$) (SA) values: 1) IOLs with negative SA to compensate for the average positive SA of the human cornea (approximately 0.27 μm) [10], 2) IOLs that correct the corneal SA leaving a slightly positive total ocular SA [4], and 3) aberration-free IOLs that are designed so that the SA of the isolated lens is corrected [11]. On one hand, several studies [4, 6, 12] have demonstrated that aspherical IOLs are more sensitive than spherical IOLs to misalignment or tilt, depending on their SA correction; i.e., the image quality in the presence of IOL misalignment is more degraded for IOL designs with a higher amount of negative spherical aberration ($Z_{4}^{0}$). However, the effect of tilt on the optical performance was less sensitive to the IOL design. On the other hand, IOLs with negative spherical aberration ($Z_{4}^{0}$) are designed with a fixed amount of negative SA to compensate for the positive SA of the average human cornea [10]. However, the corneal SA ($Z_{4}^{0}$) changes with different surgical interventions such as myopic or hyperopic refractive correction. After myopic refractive correction, corneal SA changes to a more positive value, while after hyperopic refractive correction corneal SA changes from a positive to a negative value [13]. Besides, refractive surgery increases the higher-order aberrations of the cornea, such as coma and trefoil aberrations. Regarding these possible conditions, an aberration-correction IOL, which is designed using human model corneas taken from an average of individuals, does not seem an ideal solution for those patients with prior corneal refractive surgery [14].

Several studies [15–18] have been conducted on the implantation of aspheric IOL based on preoperative corneal spherical aberration ($Z_{4}^{0}$). Al-Sayyari et al. [15] determined that a personalised aspheric IOL based on preoperative corneal spherical aberration has no significant importance comparing their results with the non-selected group. On the contrary, Beiko [17] concluded that the proper selection of the lens based on the preoperative corneal SA of the patients causes the reduction of the SA, and results in a better contrast sensitivity. Jia et al. [18] found out that personalised aspheric IOL improved mesopic contrast sensitivities at high spatial frequencies. However, few studies have investigated the corneal asphericity (Q) values in cataract patients after refractive surgeries, and its influence on visual quality following an IOL implantation.

The aim of this study is to perform a theoretical analysis of the image quality of aspheric IOLs with different amounts of SA ($Z_{4}^{0}$) with corneas with different asphericity values, to simulate myopic and hyperopic corneal refractive surgeries. IOLs are designed using Navarro´s eye model [19] and for the evaluation, two different asphericity values are applied to Navarro’s anterior corneal surface to simulate the effect of a myopic and a hyperopic corneal refractive surgery. The influence of IOL misalignment and tilt on the image quality has been assessed for the three proposed corneas and for all the IOLs under consideration.

## Materials and methods

### Eye model

To design the aspheric IOLs and to perform the evaluation of the optical quality when tilting or decentering the lenses, a numerical model of a pseudophakic eye was implemented with commercial optical design software (OSLO EDU 6.6.0, Lambda Research Corporation). The eye model was based on Navarro’s schematic eye [19] using the cornea, pupil, and retina data (see Table 1 for details). The cornea of the eye model has a refractive power of 42.16 diopters (D) and a fourth-order Zernike ($Z_{0}^{4}$) standard spherical aberration of 0.139 μm for a 6.00 mm entrance pupil diameter (5.51 mm iris diameter). The crystalline lens was replaced by the IOL. For each IOL design, the vitreous chamber depth was set in order to get the point of maximum MTF value at 100 cycles/mm for a 3.00 mm pupil diameter.

**Table 1:** Navarro’s eye model parameters used for the IOLs design and simulation.

Medium	Radius (mm)	Thickness (mm)	Refractive index at 555 nm	Conic Constant
**Anterior cornea**	7.72	0.55	1.376	-0.26
**Posterior cornea**	6.50	2.46	1.336	0.00
**Pupil**	Infinite	2.54	1.336	---
**IOL’s anterior side**	15.89	0.96	1.485	Depending on the type of aspherical surface to be designed
**IOL’s posterior side**	-13.94	18.73	1.336	Depending on the type of aspherical surface to be designed
**Retina**	-12.00			0.00

Once the different types of IOLs were designed (see more details in the Intraocular Lens Designs section), a simulation of a refractive surgery effect was carried out on Navarro's eye model cornea. Two types of refractive surgery were simulated, a myopic and a hyperopic refractive surgery, to simulate the different amounts of spherical corneal aberrations ($Z_{4}^{0}$) induced by laser ablation. The conic constants used in the post-surgery eye models were obtained from the postoperative corneal asphericity (Q) values presented in the study by Bottos et al. [20]. Table 2 shows the Q values used to simulate corneas after myopic and hyperopic laser ablation surgery and the fourth-order SA ($Z_{4}^{0}$), calculated for a 6.00 mm entrance pupil diameter with OSLO software, using only the cornea (crystalline lens was avoided), and with the retina placed at the maximum MTF position (at 100 cycles/mm for a 3.00 mm pupil). The Zernike coefficients were expressed according to the American National Standards Institute Z80.28–2017 [21].

**Table 2:** Corneal asphericity used to simulate corneas after myopic and hyperopic laser ablation surgery [20] and fourth-order SA ($Z_{4}^{0}$) calculated for a 6.00 mm entrance pupil diameter.

Cornea	Corneal anterior surface asphericity	SA ($Z_{4}^{0}$) (μm) Ø 6 mm
**A:** Normal (Navarro’s eye model cornea)	-0.26	0.139
**B:** Myopic	+0.24	0.734
**C:** Hyperopic	-0.56	-0.086

### Intraocular lens designs

The IOLs models with a refractive power of +20.00 D were designed in hydrophobic acrylic material (HF-1.2 Natural Yellow, from Benz Research & Development Corp.) with a refractive index n = 1.485 at the design wavelength *λ*_0_ = 546 nm. Three types of aspherical IOLs with anterior conical surface and one lens with spherical surfaces (-0.065 shape factor) were designed. The aspheric surfaces used to model the different IOLs take the form of a rotationally symmetric conic cross-section (see more details in Ref. [12]). Lens A was an IOL with negative fourth-order Zernike SA ($Z_{4}^{0}$) to totally compensate for the fourth-order Zernike positive SA ($Z_{4}^{0}$) of the Navarro cornea (SA = 0.139 μm at a 6.00 mm entrance pupil diameter). Lens B was designed as an IOL that does not add any fourth-order Zernike SA ($Z_{4}^{0}$) to the eye, taking into account the convergent light beam that comes from the cornea [11]. Lens C was designed with an amount of SA ($Z_{4}^{0}$) to partially correct the positive fourth-order Zernike SA of Navarro’s cornea. Table 3 shows the IOL design parameters used for the simulation and the spherical aberration of the IOL for a 6.00 mm pupil diameter.

**Table 3:** Parameters of the IOLs used in the study.

IOL	Radius Curvature (mm)		Center Thickness (mm)*	Lens Design	K (anterior surface)	SA (μm) Ø6 mm
	Anterior	Posterior				
**+20.00 D**	15.89	-13.94	0.96	**Lens A**	-30.47	-0.139
				**Lens B**	-10.91	0.00
				**Lens C**	-19.36	-0.069
				**Spherical**	0.00	+0.123

K represents the conic constant values and SA represents the spherical aberration of the IOL for a 6.00 mm pupil diameter.

*The center thickness was calculated to get 0.35 mm edge thickness at the full diameter of 6.00 mm.

### Numerical simulations

Once each IOL was designed (see Table 1), its optical performance was evaluated using OSLO optical design software for each cornea (Navarro´s eye model cornea, and modified Navarro’s eye model corneas (see Table 2)). The optical performance was evaluated for different alignment and tilt conditions. First, IOLs were decentered in the horizontal direction from 0.00 mm (on-axis) to 1.00 mm, in steps of 0.25 mm relative to the pupil axis. Secondly, the optical IOLs axis was tilted relative to the corneal optical axis with the vertex in the pupil center (from 0.00 degree to 5.00 degree, in steps of 1.00 degree). In the tilt movement, the optical vertex of the IOL first surface was always in the pupil axis. For each misalignment and tilt, tangential and sagittal MTF at 100 cycles/mm for a 3.00 mm pupil diameter were calculated, and compared to 0.43, i.e, the minimum MTF value set in ISO 11979–2 [22], to consider than a manufactured monofocal IOL offers an adequate imaging optical quality. In each situation, the root mean square (RMS) was calculated for $Z_{2}^{0}$, for $Z_{4}^{0}$ and the square root of the sum ($Z_{2}^{-2}$ and $Z_{2}^{2}$) or ($Z_{3}^{-1}$ and $Z_{3}^{1}$) squared for defocus, primary spherical aberration, astigmatism and primary coma, respectively. The optical performance assessment was carried out for all the IOLs under consideration and each cornea described in Table 2.

In addition, images of an aleatory optotype of 0.00 LogMar visual acuity (VA) were simulated using software described in Ref. [23]. The images were simulated with the retina at the maximum MTF point for a 3.00 mm pupil diameter and for a combination of misalignment and tilt, corresponding to 1.00 mm misalignment and 5.00 degrees of tilt (called worst scenario). In this condition, the Zernike aberrations coefficients (expressed in standard ANSI) were obtained with OSLO and the point spread function (PSF) was calculated as a Fourier transform of its generalized pupil function. Finally, the PSF and the paraxial image of a given optotype are convoluted to obtain the final image (see more details in Ref. [23]). All corneas were considered to make this simulation with Lenses A, B and C.

## Results

**Fig 1:**
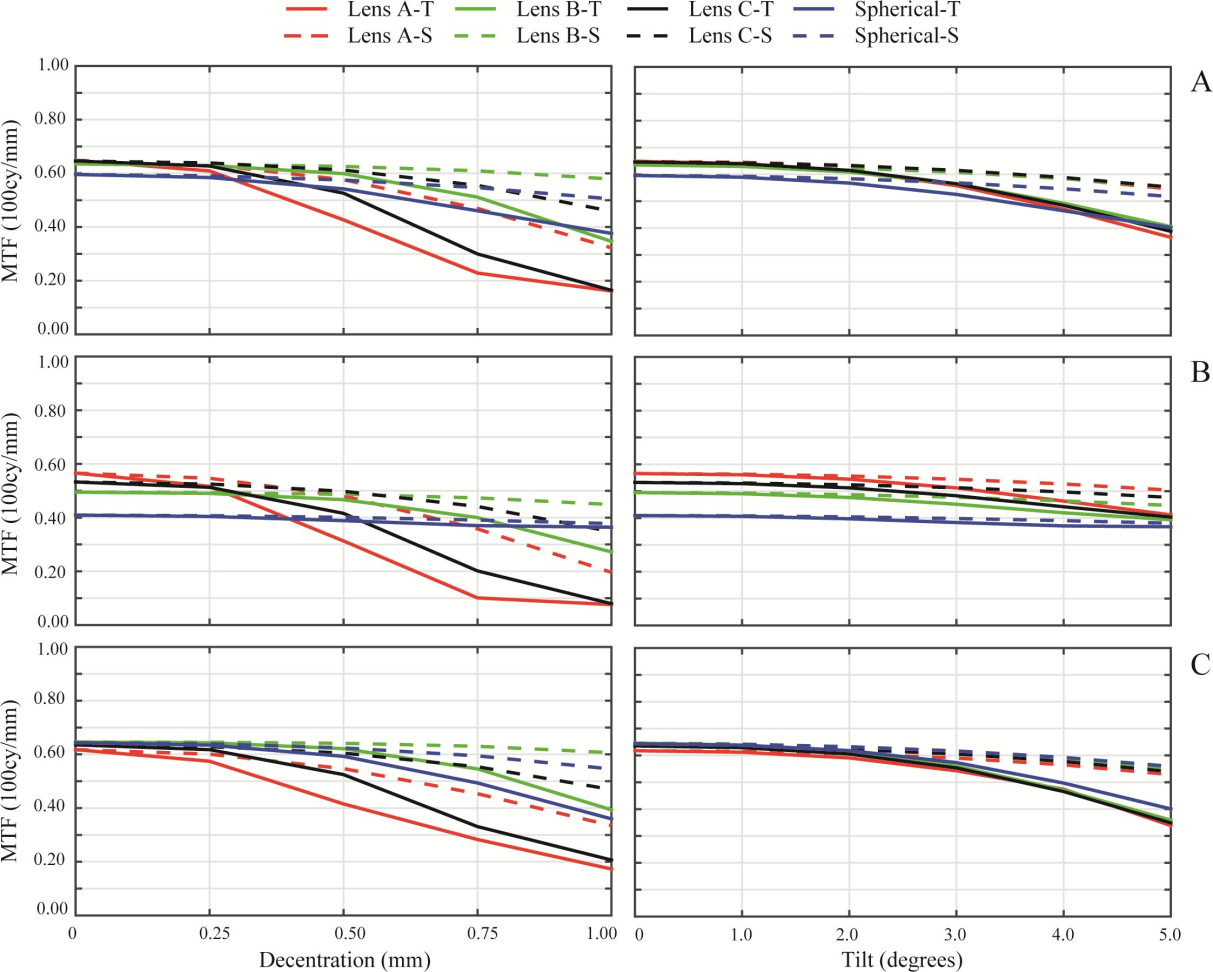
The on -axis MTF of the designed IOLs as a function of misalignment (left column) and tilt (right column) with a 3.00 mm pupil diameter and 100 cycles per degree with different corneas. The tangential MTF (continuous line) and the sagittal MTF (dashed line) are shown for each cornea: A) Navarro’s eye model cornea, B) Myopic cornea, C) Hyperopic cornea.

Fig 1 (left column) shows the MTF variation with misalignment and Fig 1 (right column) shows the MTF variation with tilt. Besides these results, Table 4 presents the maximum values of misalignment and tilt for which the average MTF values (average between tangential and sagittal values) in the different IOLs designs and corneas are below 0.43, following the tolerance limit specified in the standard ISO 11979–2 [22].

**Table 4:** Minimum values of misalignment and tilt for which the average of tangential and sagittal MTF is below 0.43 (for an object of 100 cycles/mm and a 3.00 mm pupil diameter) at every analysed cornea.

Cornea	Normal			Myopic			Hyperopic		
IOL Design	A	B	C	A	B	C	A	B	C
Misalignment (mm)	0.75	---	0.75	0.50	1.0	0.75	0.75	---	1.0
Tilt (^o^)	6.0	6.0	6.0	6.0	6.0	6.0	6.0	6.0	6.0

Note: “---” means that MTF never goes under 0.43 for the analysed misalignment and tilt ranges.

Examining the on-axis MTF values with the normal cornea (Fig 1A), Lens A offered the highest result, as it is an aberration-correcting IOL for that cornea, while for the Spherical lens the lowest value was obtained. Lens C and Lens B, in that order, had lower values than Lens A as it was expected due to their respective designs as partial aberration correction and neutral aberration IOLs. With the myopic cornea (Fig 1B), on-axis MTF values diminished for all IOL designs compared to those with the normal cornea. The Spherical lens value was the lowest while for the other designs there is not a significant difference between MTF values. With the hyperopic cornea (Fig 1C), Lens A offered the worst result, while the best on-axis MTF value was obtained for Lens B.

When the different misalignment values were applied with the normal cornea (Fig 1A), the MTF of the Lens A and C decreased rapidly to values under 0.43, for a misalignment value higher than 0.50 mm, as it is presented in Table 4. In contrast, Lens B and the Spherical lens never obtained an average MTF value under 0.43 for the studied misalignment range. Similar results were obtained with the myopic cornea (Fig 1B), with lower MTF values in general. In that case MTF values below 0.43 were obtained for Lens A and Lens C for misalignment values higher than 0.25 and 0.50 mm, respectively, while the average MTF never went under 0.43 for Lens B. With the hyperopic cornea (Fig 1C) the performance of all the IOL designs was almost similar to the results with myopic cornea once the misalignment was applied. In that cornea, Lens A and Lens C MTF values decreased under 0.43 for a misalignment higher than 0.50 and 0.75 mm, respectively. Lens B behaviour with the hyperopic cornea is comparable to the one obtained with the myopic cornea, with an MTF value always higher than 0.43.

Fig 1 (right column) shows the MTF variation with tilt. The average MTF value is never under 0.43 for any IOL design with any analysed cornea for the chosen range. The threshold tilt value has been calculated and included in Table 4. In this situation, MTF was less sensitive to the IOL design than for misalignment. With the myopic cornea (Fig 1B), the Spherical IOL is almost tilt independent. The rest of the IOL designs showed the same behaviour with that cornea but the overall MTF values are limited by the on-axis value. For the normal cornea (Fig 1A) and hyperopic cornea (Fig 1C), the Spherical lens is tilt dependent and its optical quality decreased with tilt. For these two corneas, all IOL designs followed practically the same behaviour, influenced by the on-axis MTF value.

**Fig 2:**
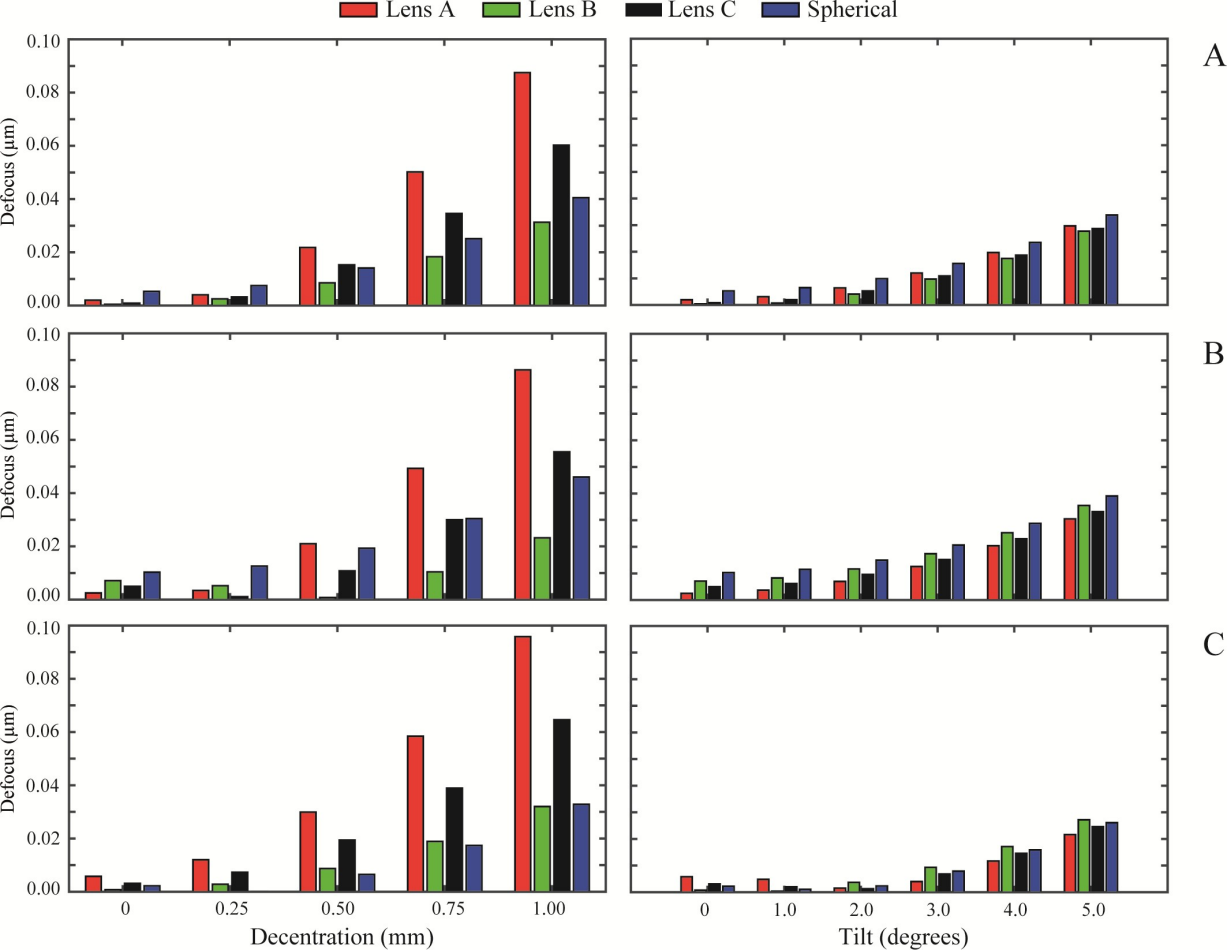
RMS defocus ($Z_{2}^{0}$) values of the designed IOLs for misalignment (left column) and tilt (right column) with each different cornea: A) Normal, B) Myopic, C) Hyperopic.

**Fig 3:**
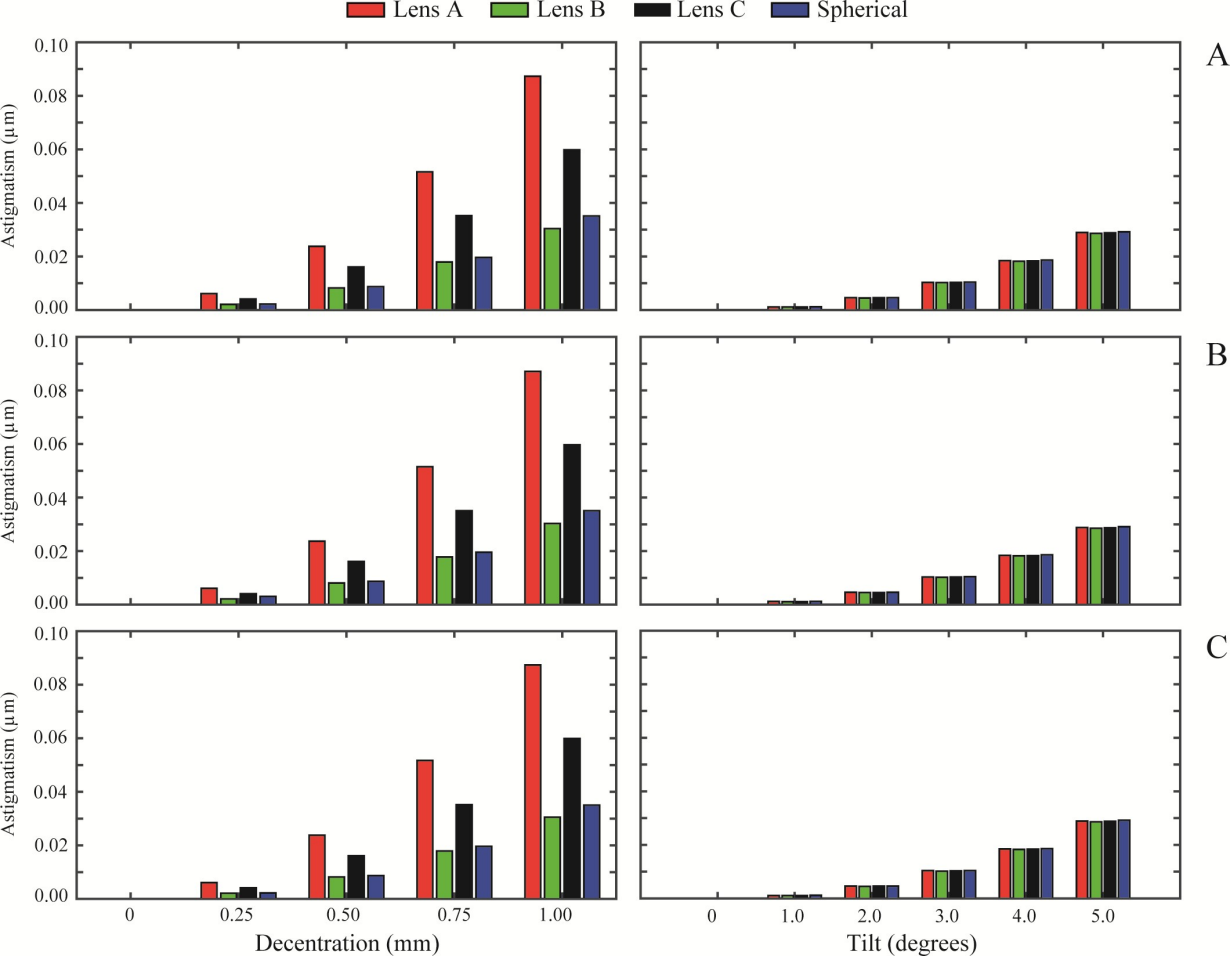
RMS astigmatism ($Z_{2}^{-2}$ and $Z_{2}^{2}$) values of the designed IOLs for misalignment (left column) and tilt (right column) with each different cornea: A) Normal, B) Myopic, C) Hyperopic.

**Fig 4:**
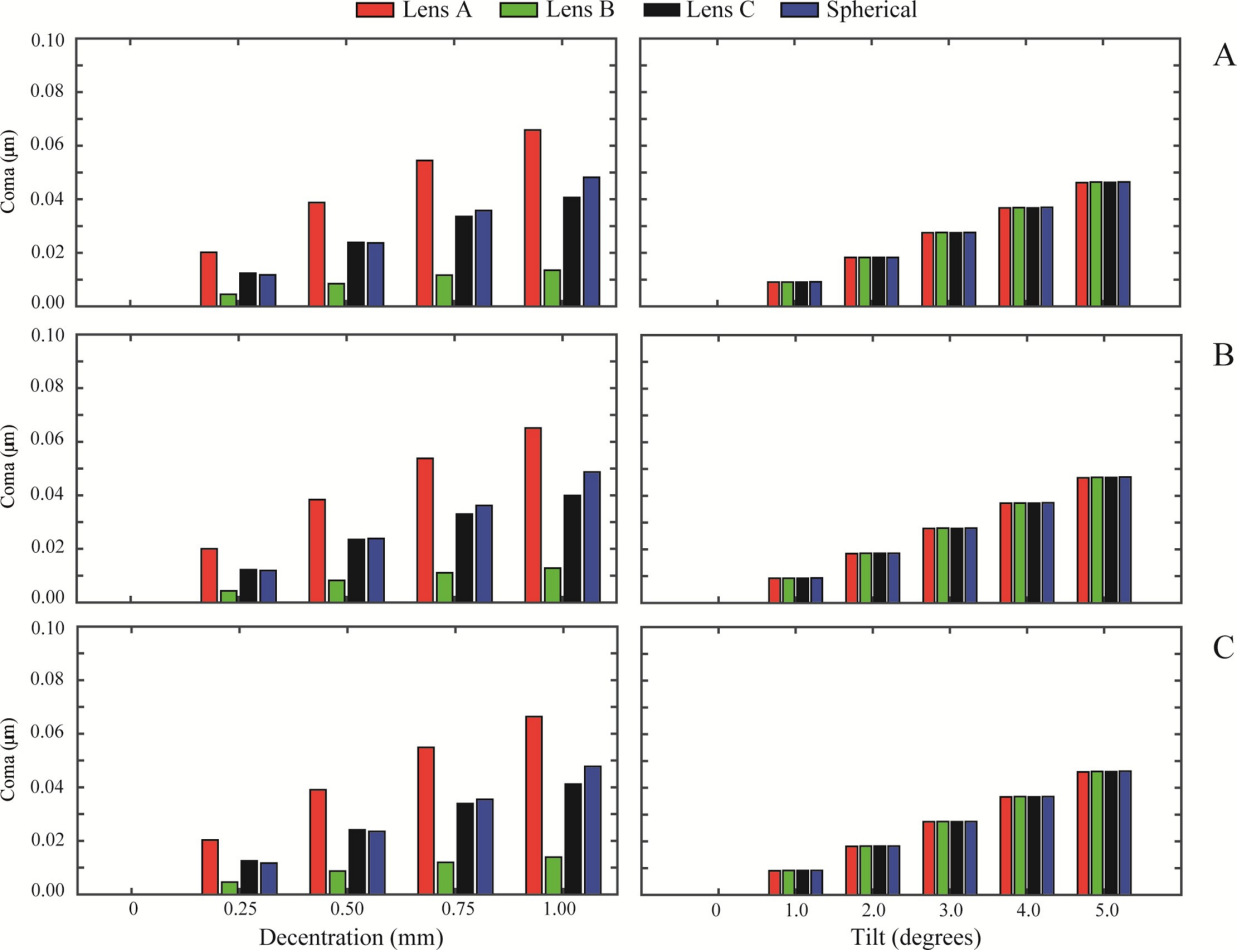
RMS coma ($Z_{3}^{-1}$ and $Z_{3}^{1}$) values of the designed IOLs for misalignment (left column) and tilt (right column) with each different cornea: A) Normal, B) Myopic, C) Hyperopic.

**Fig 5:**
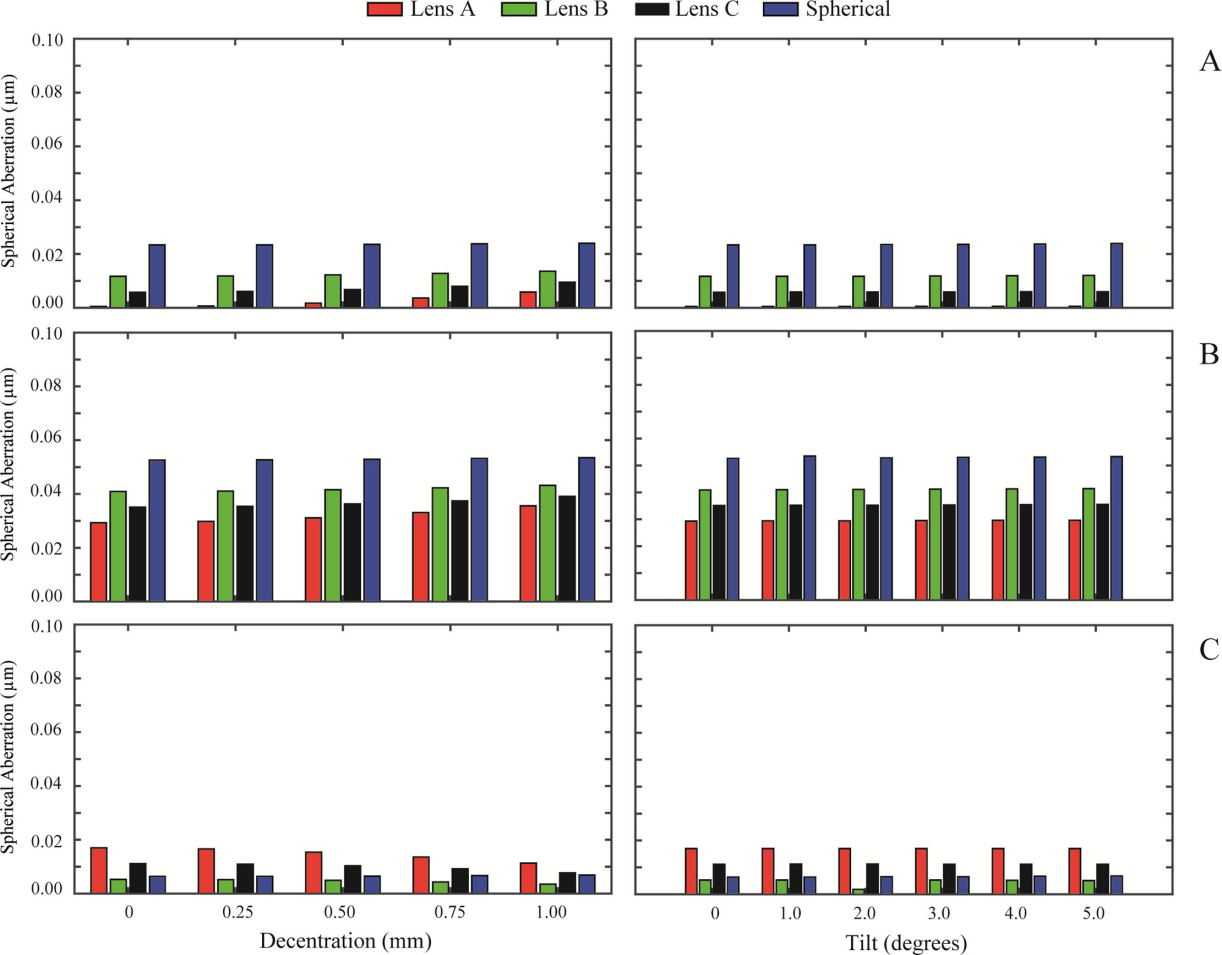
RMS spherical aberration ($Z_{4}^{0}$) values of the designed IOLs for misalignment (left column) and tilt (right column) with each different cornea: A) Normal, B) Myopic, C) Hyperopic.

Fig 2A, 2B and 2C show the RMS defocus results for the normal cornea, myopic cornea, and hyperopic cornea, respectively. The same layout is followed in the results of RMS astigmatism, in Fig 3, for RMS coma in Fig 4 and for primary spherical aberration ($Z_{4}^{0}$) in Fig 5. As an overview, independently of the IOL design, a misalignment or a tilt increases the wavefront aberrations at any studied cornea. RMS values for astigmatism and coma are zero when the IOL is located on axis. In Fig 2 (left column), Lens A induced the highest RMS defocus value from a 0.50 mm misalignment and onwards, for any analysed cornea. In contrast, Lens B offered the lower RMS defocus results with misalignment. For tilt values (Fig 2 right column), the variation in the RMS defocus is small in all the IOLs designs analysed for the three corneas. A similar behaviour occurred for RMS astigmatism (Fig 3) and for RMS coma (Fig 4). Nevertheless, it is for the RMS coma where Lens B offers the best results for misalignment, compared with the other IOL designs.

Fig 5A, 5B and 5C show the RMS Spherical aberration results for the normal cornea, myopic cornea, and hyperopic cornea, respectively. Results show that primary SA is maintained in all lenses with the three corneas, for all misalignments (left column) and tilts (right column). The on-axis value is preserved without practically variations. Note, that SA ($Z_{4}^{0}$) in the myopic eye (Fig 5B) is higher than SA in normal and hyperopic eyes (Fig 5A and 5C). This is because of the the fourth-order SA ($Z_{4}^{0}$), calculated for a 6.00 mm entrance pupil diameter (see Table 2) is higher in this type of cornea.

**Fig 6:**
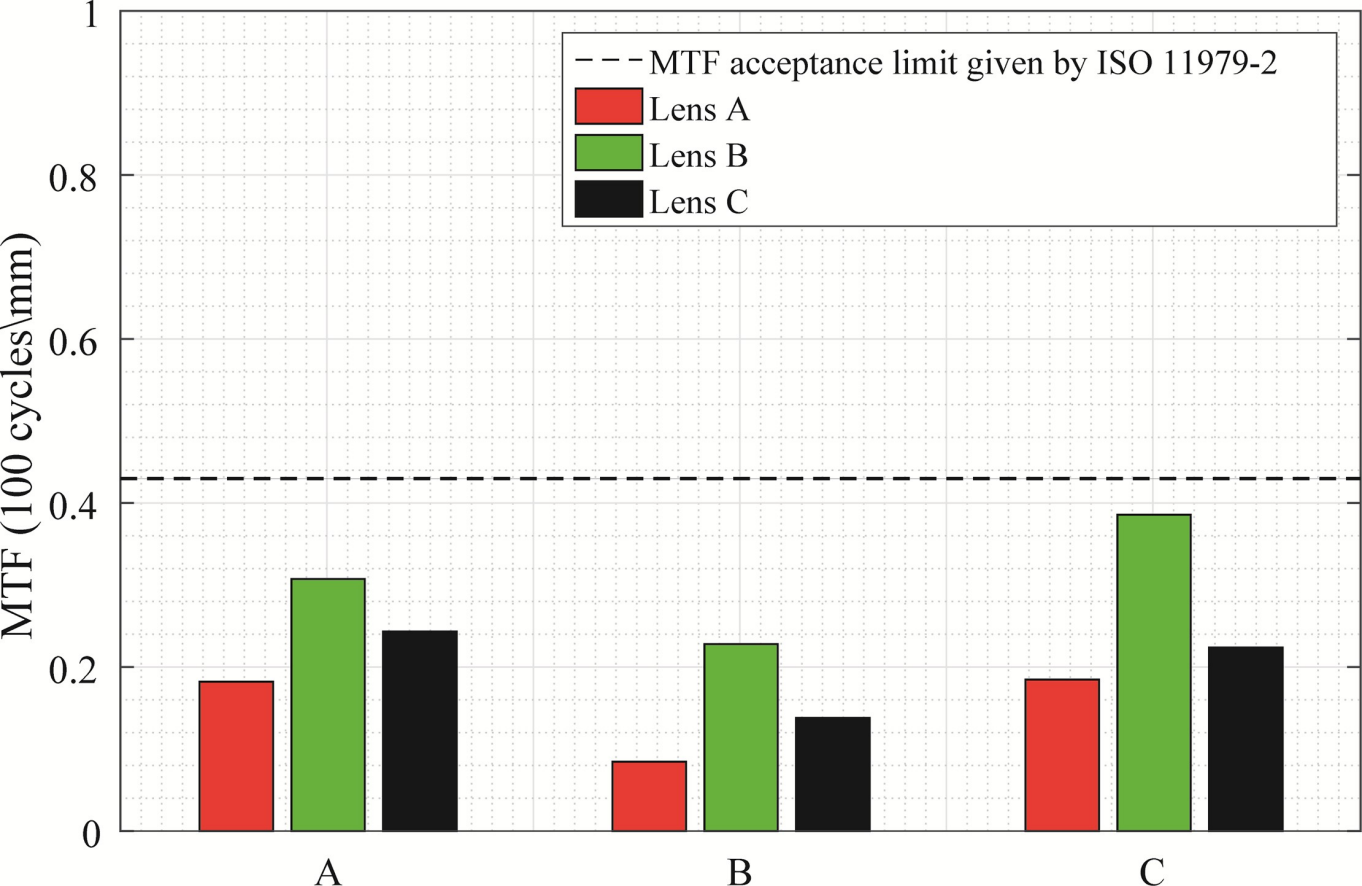
**MTF average values (average between tangential and sagittal) for each IOL design (Lens A, Lens B and Lens C) with each cornea, in the worst-case scenario: 5 degrees of tilt and 1 mm misalignment.** Corneas are represented in rows: A) Normal, B) Myopic, C) Hyperopic.

In Fig 6 the average MTF values for Lenses A, B and C at the worst-case scenario (tilted 5.00 degrees and with a misalignment of 1.00 mm) with the three corneas, for a 3.00 mm pupil diameter are presented. For none of the IOL designs, an average MTF above 0.43 is obtained with any considered cornea. For all corneas, the average MTF values followed the same tendency. For the normal cornea (Fig 6A), the highest MTF value was obtained for Lens B, with 0.307 as the average MTF, followed by Lens C with an average MTF of 0.243. Lens A was the IOL with the lowest MTF with the normal cornea (0.182 as the average MTF). For the myopic and hyperopic corneas (Fig 6B and 6C), the highest MTF value was obtained for Lens B, while Lens A remained as the IOL with the lowest MTF. With the myopic cornea, all lenses had their MTF values reduced. However, it is remarkable how with the hyperopic cornea Lens B MTF increased to a value of 0.386.

**Fig 7:**
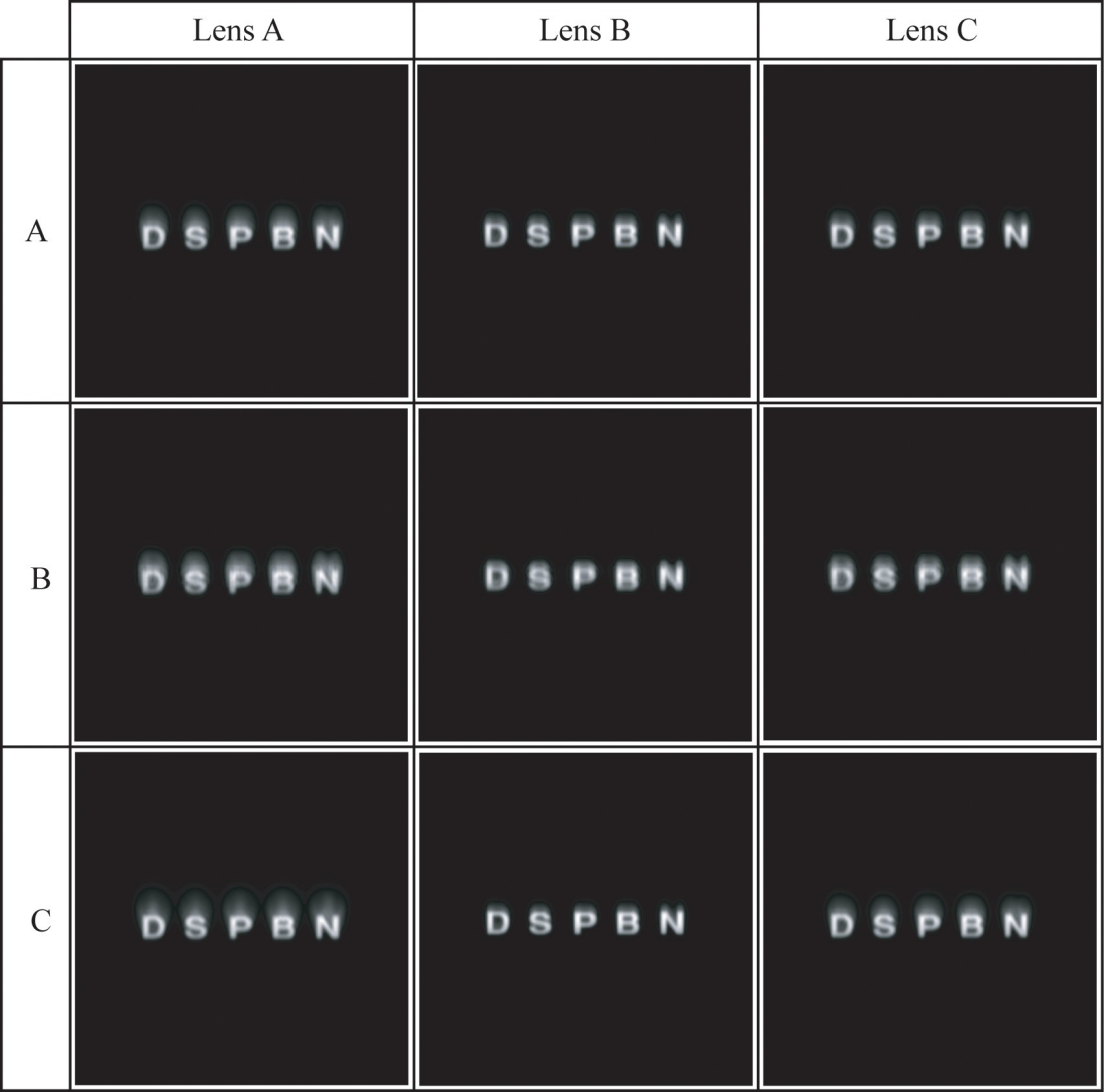
Simulation of the retinal images of a 5’ angular size optotype (VA of 0.00 LogMar scale) generated by each designed IOL (in columns: Lens A, Lens B and Lens C) with every cornea (in rows: A) Normal, B) Myopic, C) Hyperopic) at the worst-case scenario (a combination of 5 degrees of tilt and 1 mm misalignment of the IOL).

The results shown in Fig 6 are in concordance with the visual simulation images in Fig 7, where the theoretical visual response of Lenses A, B and C in the worst-case scenario for a 3.00 mm pupil diameter with the three corneas is shown. Using the results with the normal cornea as a reference (row A in Fig 7), the worst visual results were obtained for the myopic cornea (row B in Fig 7), as it was predicted by the MTF deterioration with this cornea in Fig 6. With the hyperopic cornea (row C in Fig 7), visual results were better for all lenses compared with the myopic cornea, and for Lens B, the visual result with the hyperopic cornea (see the Lens B column at the row C, in Fig 7) was even preferable than the result in the normal cornea.

## Discussion

Nowadays, cataract surgery not only replaces an opacified crystalline lens from the eye with an artificial IOL, but it also seeks for spectacle independence of the patient and the highest possible optical quality. To guarantee the acquisition of the maximum optical quality with premium lenses designs [24], it is crucial to ensure proper IOL stability inside the eye. Some studies reported an appearance of astigmatism and Higher Order Aberrations (HOAs) such as coma with IOL misalignment [4] and tilt [25, 26]. IOL stability inside the eye is influenced by many factors such as the capsular bag diameter related to the IOL overall diameter [27], the haptic design and lens material [7], the surgical technique [28], and even some postoperative actions like the Nd:YAG laser application for posterior capsulotomy [29].

Several studies demonstrate that aspheric IOLs with aberration-free designs avoid optical quality loss when a misalignment occurs [12]. However, IOLs are commonly designed using theoretical eye models by applying aspherical surfaces to the IOL [10, 30]. A major problem is that these theoretical eye models use average biometry from a large population that might not match the particular anterior corneal radius and asphericity of an ablation surgery patient. After refractive surgery, SA changes to more positive values in case of myopic correction whereas it turns into negative ones in case of hyperopic correction [31, 32].

The aim of this study is to perform a theoretical analysis of the image quality of three types of aspheric IOLs designed with different amounts of SA using Navarro´s eye with different corneal asphericity values. Myopic and hyperopic eyes were generated by changing the eye model corneal asphericity to +0.24 and -0.56 respectively, according to the results by Bottos et al. [20]. We considered 5 misalignment values (ranging from 0.00 mm, on-axis, to 1.00 mm; in 0.25 mm steps) and 6 tilt values (from 0.00 degrees, on-axis, to 5.00 degrees; in 1.00 degrees steps), in agreement with previous studies [5, 12, 33]. However, the heterogeneity of experimental methods such as Scheimplug imaging, Purkinje reflections, optical coherence tomography, and slit lamp assessment to measure IOL misalignment or tilt once it has been implanted inside a pseudophakic eye make results comparison difficult. Similar values to those in our study have been reported in other clinical studies [34–36], where the typical mean misalignment is 0.30±0.16 mm [a range from 0.00 mm to 1.09 mm] and the mean tilt is 2.62±1.14 degrees [a range from 0.20 to 8.17 degrees]. However, IOL decentration and tilt can be different than these values in various clinical situations [37–39]. For example, Wang et al. [37] obtained an average of 4.9 ± 1.8 degrees [a range from 1.6 to 10.7 degrees] and Leisser et al. [38] reported a mean tilt of 4.1 ± 1.9° and mean decentration of 0.31 ± 0.14 mm, 2 months after surgery with a Purkinje meter.

To our knowledge, this is the first study where aspheric IOLs with different amounts of SA designed for a particular theoretical eye model are studied together with corneas with extreme corneal asphericities, applying some misalignment and tilt errors. Beiko [17] and Jia et al. [18] studied the implantation of personalised IOLs according to the corneal SA ($Z_{4}^{0}$), but in this case, we studied the consequences of implanting an IOL designed for a different amount of SA, which is related to the corneal asphericity.

We found out that the optical quality, evaluated by means of the MTF, is dependent on the amount of SA correction of the IOLs for every studied cornea (see Figs 1 and 6). Besides this finding, the performance of the different IOL designs in terms of optical quality is the same when comparing all the corneas. Lenses A, B and C follow the same tendency with all evaluated corneas, varying the total amount of MTF at each misalignment or tilt. Using as reference the on-axis MTF values obtained for Navarro’s eye model, results with the myopic cornea were lower for all the IOL designs. Regarding the optical quality behaviour of the lenses with tilt, we observed what it was known already: MTF degradation was less dependent on the IOL design [12]. The tendency followed in the MTF degradation with tilt was similar for all lenses, varying the initial on-axis MTF value in relation to the studied cornea.

Results also show that IOL misalignment and tilt increase wavefront aberrations for every cornea (see Figs 2, 3 and 4), except for the primary spherical aberration (Fig 5), which is almost stable. As might be expected, there is no astigmatism or coma in the on-axis position of the IOLs. Lens A produces the larger increase in the HOAs for misalignments greater than 0.25 mm. This was also found by Pérez-Merino et al. [4], in their study there was an increase in coma and astigmatism with a misalignment of 0.7 mm for all studied IOLs. And the magnitude of the increase depends on the amount of SA correction produced by the IOL. The relation between tilt and the appearance of HOAs is less dependent on the IOL design, presenting all lenses a similar behaviour for all the corneas (see Figs 2, 3 and 4, right columns). Moreover, as can be seen in Figs 3 and 4, respectively, astigmatism and coma are small but not zero for a decentered lens B despite the fact that it was designed free of 4^th^-order spherical aberration $Z_{4}^{0}$. This particular behavior is due to the influence that higher order spherical aberration (higher than fourth) has in a decentered position.

Results also show that the RMS for primary SA $Z_{4}^{0}$ is maintained for all IOL design with misalignment and tilt in the three corneas. This behaviour of a constant SA can be correlated with the findings of the study of Wang et al. [40], where the SA does not change in an optical system with a misalignment in the pupil.

In a previous study [12], we found that Lens B was the lens which preserved best its optical quality, in terms of MTF and HOAs, against misalignment and tilt. Here, the variation of the corneal asphericity of Navarro’s eye model also reveals that Lens B offers a better optical quality for a myopic and hyperopic cornea.

In Fig 6, where the MTF values at the worst-case scenario are shown (lenses with 5.00 degrees tilt and 1.00 mm misalignment) for all the corneas, can be seen that for Lens B the higher MTF values are obtained. These findings are supported by the visual simulation in Fig 7. The image of the chart for Lens B with the hyperopic cornea is even better than with the normal cornea. A similar finding was obtained in the study by Mckelvie et al. [41], who stated that using three IOLs presence in the market and a purpose-built physical model eye, the IOLs with SA closer to zero, or zero (aberration-free IOL), maintained best the optical quality against misalignments and tilts.

In conclusion, it was proved that, regarding the MTF, HOAs, and visual simulation analysis, an IOL initially designed to not add any fourth-order Zernike SA $\left(Z_{4}^{0}\right)$ to the eye provides a better optical performance than an IOL designed to compensate, totally or partially, the fourth-order Zernike SA of the cornea when there is a misalignment in an eye with any value of corneal asphericity. Designing IOLs according to the patient’s corneal SA is a good approach to improve the optical quality after the implantation, because what Al-Sayyari et al. [15] and Solomon et al. [42] found is that there is no significant change in the corneal SA after a cataract surgery. It is important to note that despite the fact that we are dealing with a typical average in-the-bag axial IOL position (2.54 mm after the iris plane) it was also checked that even extremal ±1.5 mm changes around this selected IOL axial position have no influence on the results obtained in this paper.

Considering the limitations of the study, the authors know that the HOAs of the cornea are not only composed of SA ($Z_{4}^{0}$), but also aberrations such as coma, trefoil and high-order aspheric terms are considered as HOAs [43]. These aberrations could not be neglected due to their contribution to the final image quality. Therefore, the implantation of personalised IOLs to correct HOAs of an individual cornea would be an interesting issue for future clinical and scientific research.

It is important to note that the results of this study can only be applied to situations where our cornea model and the refractive surgery technique (LASIK) were used. New surgical techniques, as SMILE and FLEx, can induce smaller spherical aberration changes than LASIK [44] so the results of this study could not be applied to them. Moreover, regarding the post-LASIK model cornea used in this work, it is important to remark that despite the fact than it corresponds to an early surgery stage (1 to 3 months after surgery), it is not very risky to assume, attending to current evidence as Vega-Estrada et al [45], that it can also be suitable for a more advanced state previous to an IOL replacement intervention. Similar results were reported in some other clinical studies [46–49].

For the sake of simplicity in this study the aspherical lenses were designed with pure conical surfaces. However, due to the fact that other functional form can be used to describe them [50, 51] it is important to note that this conical condition implies also a relative limitation to the reach of this work results because slightly different values could be obtained for other aspherical design strategies.

## Supporting information

S1 File MTF of the designed IOLs as a function of misalignment and tilt for each cornea. (XLSX)

S2 File Results of RMS defocus for decentration and for tilt with each different cornea. (XLSX)

S3 File Results of RMS astigmatism for decentration and for tilt with each different cornea. (XLSX)

S4 File Results of RMS coma for decentration and for tilt with each different cornea. (XLSX)

S5 File Results of RMS spherical for decentration and for tilt with each different cornea. (XLSX)

S6 File MTF average values for each IOL design with each cornea, in the worst-case scenario. (XLSX)
